# Recommendations for Reporting Alcohol, Tobacco, and Other Drug Screening Tool Use With Pregnant Aboriginal and Torres Strait Islander Peoples

**DOI:** 10.1002/hpja.70103

**Published:** 2025-09-22

**Authors:** Christina A. Norris, Tahlia Johnson, Ashlea Bartram, Armin Muminovic, Alice McEntee, Jane Fischer, Alison Francis, Jacqueline A. Bowden

**Affiliations:** ^1^ National Centre for Education and Training on Addiction Flinders University Adelaide Australia; ^2^ Flinders Health and Medical Research Institute, College of Medicine and Public Health Flinders University Adelaide Australia; ^3^ College of Nursing and Health Sciences Flinders University Adelaide Australia; ^4^ Drug and Alcohol Services South Australia Adelaide Australia

**Keywords:** Aboriginal and Torres Strait Islander, alcohol, pregnancy, screening, tobacco

## Abstract

Alcohol, tobacco and other drug screening tools are essential components of health promotion strategies to help identify individuals at risk of harmful substance use and guide them towards timely and appropriate interventions. These tools play a particularly important role in antenatal care, where routine screening for pregnant women supports healthier pregnancies and promotes long‐term wellbeing for mother and child. However, it is unclear whether there are culturally responsive, validated tools for use with pregnant Aboriginal and Torres Strait Islander peoples. A search of recent literature identified tools commonly used with Aboriginal and Torres Strait Islander populations but found limitations in how the use of these tools was reported. We outline key considerations for improving the implementation and reporting of alcohol, tobacco and other drug screening tools in Aboriginal and Torres Strait Islander populations. We discuss how prioritising cultural responsiveness, validation and acceptability in screening practices can enhance healthcare equity and improve outcomes for pregnant Aboriginal and Torres Strait Islander peoples affected by alcohol, tobacco, and other drug use.

Alcohol, tobacco and other drug (ATOD) screening tools are used to identify people at risk of harmful substance use that may lead to health or social harms and who might benefit from intervention [1]. These tools are particularly useful in antenatal care, where ATOD screening is routinely conducted for pregnant women [2] and aligns with global strategies such as the World Health Organization's SAFER initiative, which promotes evidence‐based interventions to reduce alcohol‐related harm through access to screening, brief intervention and treatment. While ATOD use during pregnancy increases the risk of poor maternal health and poor birth outcomes [3], it remains unclear if culturally responsive, validated tools exist for use with pregnant Aboriginal and Torres Strait Islander peoples. Pregnancy is a critical time to support maternal well‐being and promote positive birth outcomes. Culturally responsive screening that acknowledges the strengths and resilience of these communities can enhance maternal and child health [4]. The National Guide to a Preventive Health Assessment for Aboriginal and Torres Strait Islander People, developed by the RACGP and NACCHO, recommends that all pregnant Aboriginal and Torres Strait Islander women be routinely screened for smoking, alcohol and other drug use at the first antenatal visit and throughout pregnancy. This reinforces the need for culturally responsive, validated tools to support this recommendation and ensure screening is conducted in a safe and acceptable manner [5].

Validated ATOD screening tools for non‐Aboriginal people exist; however, their cultural suitability for pregnant Aboriginal and Torres Strait Islander peoples remains unclear. In May 2023, the National Centre for Education and Training on Addiction conducted a review of literature published from 2018 to 2023 to identify ATOD screening tools commonly used with pregnant Aboriginal and Torres Strait Islander peoples [6]. The review included a systematic search of five electronic databases (Medline, CINAHL, PsycINFO, Scopus, and Informit) and consultation with key Australian organisations (alcohol and other drug peak bodies, Primary Health Networks, and Aboriginal Community Controlled Health Organisations). We sought to identify evidence on the cultural responsiveness and validation of commonly used ATOD screening tools. However, we found that none of the recent studies in the review provided evidence of using screening tools that had been validated for use with pregnant Aboriginal and Torres Strait Islander peoples or that were culturally responsive or assessed for acceptability. This highlights a critical gap in the evidence base regarding the use of culturally appropriate ATOD screening tools in antenatal care.

ATOD screening tools cannot be directly transferred for use from non‐Aboriginal to Aboriginal and Torres Strait Islander peoples [7], or transferred to pregnant Aboriginal and Torres Strait Islander peoples without addressing psychosocial complexities [8]. Aboriginal and Torres Strait Islander children are disproportionately represented in out‐of‐home care compared to non‐Aboriginal children [9]. Intergenerational trauma and the risk of child removal further highlight the need for culturally responsive, validated tools. Therefore, we have a unique opportunity to highlight the importance of recognising the need for culturally responsive and validated ATOD screening tools for pregnant Aboriginal and Torres Strait Islander peoples. This includes adapting the tools to be culturally responsive and acceptable to each diverse community. Using culturally responsive and appropriate ATOD screening tools can reduce stigma, support disclosure of substance use, improve child protection outcomes, and enhance engagement with antenatal care among Aboriginal and Torres Strait Islander peoples.

Improving the cultural responsiveness, acceptability, and validation of ATOD screening tools aligns with health promotion principles of equity, empowerment, and cultural safety and reflects increasing calls for community‐led, strengths‐based approaches to care delivery [10]. Short, culturally responsive, non‐judgemental screening tools are preferred for initial and routine screening [11]. This commentary outlines the ATOD screening tools we identified as most commonly used with pregnant Aboriginal and Torres Strait Islander peoples, offering recommendations for future reporting, based on challenges experienced when conducting our evidence review [6].

## 1 Alcohol, Tobacco and Other Drug Screening Tools Commonly Used With Pregnant Aboriginal and Torres Strait Islander Peoples

### 1.1 Alcohol Use Disorders Identification Test (AUDIT) [12] and AUDIT–Consumption (AUDIT‐C) [13]

Our evidence review [6] found the AUDIT and AUDIT‐C were the most the commonly used screening tools with pregnant Aboriginal and Torres Strait Islander peoples. The AUDIT is an internationally validated 10‐item screening tool developed by the World Health Organization, which screens for risky alcohol consumption and potential dependence [14]. The AUDIT‐C comprises the three alcohol consumption items and can identify individuals with risky alcohol use or alcohol use disorders. The AUDIT‐C has been used with Aboriginal and Torres Strait Islander peoples in various settings, including its well‐established use in Aboriginal Community Controlled Health Organisations [15, 16]. However, like the AUDIT, it has not been specifically validated for use in antenatal care with pregnant Aboriginal and Torres Strait Islander peoples. The AUDIT‐C is embedded within the Grog Survey App, which collects data on alcohol consumption in a culturally responsive way. The Grog Survey app is an interactive and visual tool administered via a tablet computer application and was developed through an iterative consultation process with Aboriginal and Torres Strait Islander knowledge holders and clinical experts [17]. It has been found to be valid and appropriate for use in clinical settings [18], but not specifically with pregnant Aboriginal and Torres Strait Islander peoples.

### 1.2 Alcohol, Smoking, and Substance Involvement Screening Test (ASSIST) [19]

Developed by the World Health Organization by an international group of addiction researchers and clinicians, the ASSIST is an 8‐item screening tool that aids in the early identification of substance use‐related health risks and substance use disorders in a primary healthcare setting [20]. The ASSIST has been validated with Aboriginal and Torres Strait Islander peoples [21], although not specifically with pregnant Aboriginal and Torres Strait Islander peoples. Subsequent to the release of the original ASSIST, a recent study has detailed first‐stage development of the Pitjantjatjara translation of the ASSIST [21], suggesting potential for broader utilisation in the Pitjantjatjara‐speaking population, potentially including pregnant Aboriginal and Torres Strait Islander peoples.

## 2 Recommendations for Reporting Use of Alcohol, Tobacco and Other Drug Screening Tools

To address some of the challenges and build the evidence base, future research involving ATOD screening of pregnant Aboriginal and Torres Strait Islander peoples should, wherever practicable, report details of the cultural responsiveness, acceptability and validation of the tools used within the target population.

### 2.1 Cultural Responsiveness

Cultural responsiveness describes actions that create and maintain cultural safety, focusing on embedding cultural understanding [22]. Additionally, cultural responsiveness emphasises knowing, being, and doing—acknowledging culture as central to people's experiences and actively engaging with and adapting practices to meet the cultural needs of individuals and communities [22]. Cultural responsiveness is driven by effective community‐led engagement and high levels of community oversight [22]. As every community has idiosyncratic norms, languages and practices, this could be achieved by effective co‐design with individual communities [10]. Additionally, targeted training for healthcare staff to administer ATOD screening tools has shown promise in improving cultural sensitivity and screening rates [23].

### 2.2 Acceptability

Acceptability assesses how well an intervention is received by the target population [24]. Establishing acceptability in terms of ATOD screening tools involves assessing whether the tool meets the expectations, needs, preferences, and standards of the intended audience. Common methods to help determine acceptability include qualitative methods [25]. Common questions include asking about personal relevance, appropriateness and barriers [26]. Acceptability studies aim to understand the target population's perceptions, experiences and attitudes towards the intervention to assess specific acceptability outcome measures [24].

### 2.3 Validation

Validation of ATOD screening tools with a specific population helps improve accuracy, relevance, effectiveness, and generalisability with the target population. Validation of a tool clarifies whether findings are valid, ultimately leading to better outcomes for the intended audience. Validation of a screening tool is a process often involving comparison between measures and testing against other previously validated tools [18]. Validating ATOD screening tools for pregnant Aboriginal and Torres Strait Islander peoples involves testing that the tools accurately measure what they are intended to measure within the cultural context of these populations [14]. However, screening tools that have never been validated can still be valid.

## 3 Implications for Policy and Research

As previously mentioned, the World Health Organization's SAFER initiative reinforces the importance of routine alcohol screening and brief intervention as part of a comprehensive public health strategy to reduce alcohol‐related harms, highlighting the need for continued implementation and evaluation in antenatal and broader healthcare settings [27]. There is evidence demonstrating the value and feasibility of culturally grounded tool development in partnership with Aboriginal and Torres Strait Islander communities. For example, a recent study used Indigenous‐led methods to develop and validate a strengths‐based mental health and wellbeing scale, demonstrating an effective model for culturally safe and collaborative tool design [28]. Similar approaches should be considered in the development of ATOD screening tools. Another example is the Kimberley Mum's Mood Scale (KMMS), a culturally adapted perinatal mental health screening tool co‐designed with Aboriginal women and health professionals, demonstrating that culturally responsive, validated, and acceptable screening tools can be developed and successfully implemented in antenatal care [29]. The KMMS has undergone validation and acceptability testing specifically with pregnant Aboriginal and Torres Strait Islander women, providing a model for similar approaches to screening for ATOD use in pregnancy [5]. This example reinforces the need for investment in co‐designed, culturally appropriate ATOD screening tools.

Embedding cultural responsiveness into screening practices aligns with the Ottawa Charter for Health Promotion, which calls for health promotion to “create supportive environments” and “strengthen community action [30].” These principles are particularly relevant in antenatal care settings, where culturally safe approaches can influence intergenerational health outcomes. While validated ATOD screening tools exist for use with Aboriginal and Torres Strait Islander peoples, none of the studies included in our review reported acceptability, validation, or cultural responsiveness for use with pregnant Aboriginal and Torres Strait Islander peoples. Improving reporting of ATOD screening tools in research may contribute to the development of more equitable and culturally responsive screening interventions and research practices, ultimately improving health outcomes.

Health promotion research highlights the importance of embedding cultural responsiveness within broader structural and service‐level change. For example, a scoping review found that many health promotion programmes targeting Aboriginal and Torres Strait Islander communities focused on individual behaviour while overlooking the importance of supportive environments and culturally grounded implementation strategies [31]. Similarly, another study emphasised that factors such as leadership support, culturally competent staff, and long‐term community partnerships are critical to enabling the reorientation of health services in line with health promotion principles [32].

Ongoing adaptation of ATOD screening tools for diverse populations, including pregnant Aboriginal and Torres Strait Islander peoples, could further strengthen their utility and alignment with such principles. By prioritising cultural responsiveness, acceptability and validation in screening practices, progress can be made towards improving the health outcomes and well‐being of pregnant Aboriginal and Torres Strait Islander peoples. To support this, studies should routinely report on the cultural responsiveness, acceptability and validation of ATOD screening tools used with pregnant Aboriginal and Torres Strait Islander peoples.

## Ethics Statement

The authors have nothing to report.

## Conflicts of Interest

The authors declare the following financial interests/personal relationships which may be considered as potential competing interests: Christina A. Norris; Ashlea Bartram; Alice McEntee; Jane Fischer; Alison Francis and Jacqueline A. Bowden report financial support was provided by Drug and Alcohol Services South Australia. Christina A. Norris; Ashlea Bartram; Armin Muminovic; Alice McEntee; Jane Fischer and Jacqueline A. Bowden report financial support was provided by the Australian Government Department of Health and Aged Care. All authors declare they have no known personal relationships that could have appeared to influence the work reported in this paper.

## Data Availability

Data sharing not applicable to this article as no datasets were generated or analysed during the current study.
